# Beyond the first 25 years: The International AIDS Society and its role in the global response to AIDS

**DOI:** 10.1186/1742-4690-3-85

**Published:** 2006-12-01

**Authors:** Pedro Cahn, Craig McClure

**Affiliations:** 1 International AIDS Society, 33, Chemin de l'Avanchet, Geneva, Switzerland

## Abstract

Dr. Pedro Cahn, International AIDS Society (IAS) President and Mr. Craig McClure, IAS Executive Director, provide their thoughts and analysis on the current and future role of the IAS as part of the global response to HIV/AIDS.

## Commentary

In 1981 the first indication of a disturbing new illness that defied medical classification appeared in the Center for Disease Control and Prevention's Morbidity and Mortality Weekly Report [1]. Little could have prepared the scientific community – or the world – for the human catastrophe that followed. We are now just past the twenty-five year mark in the history of this epidemic and yet, despite impressive scientific advances in both diagnosis and treatment, it is continuing to outpace our efforts to contain it. This year marked a number of anniversaries in our history with this epidemic, including 10 years since the advent of highly active antiretroviral therapy (HAART), so it is a useful point at which to reflect on our progress with this disease and to chart a way through the difficult terrain ahead.

As the world's leading independent association of professionals working in HIV, the International AIDS Society (IAS) has a unique role to play as part of the global response to this epidemic in the decades to come. With over 10,000 members from 171 countries, the IAS represents individuals working at every level of the response, from scientists and clinicians to policymakers and community educators. The IAS was established as a non-profit organization in 1988, with a mandate to organize the biennial International AIDS Conferences, although the Stockholm-based staff remained small and responsibility for organizing the conferences was primarily undertaken by professional conference organizers. That changed in 2004, when the Governing Council of the IAS decided to upgrade its structure by strengthening the professional staff at the secretariat and moving the headquarters to Geneva, Switzerland. The move was intended to strengthen links with other health NGOs and UN multilateral agencies. Since then the IAS secretariat has grown to a professional staff of 30, and the Governing Council has established an ambitious agenda of work for the organization over the coming years, outlined in Stronger Together: Strategic Framework 2005 – 2009, available on the IAS website [2].

An initial priority for the IAS in 2005 was to undertake a comprehensive review of the International AIDS Conference, which had evolved from a small medical meeting in Atlanta in 1984 to a massive, multisectoral health conference attended by over 15,000 participants in Bangkok in 2004, and over 20,000 in Toronto in 2006. The IAS consulted broadly with stakeholders on how to maximize the reach and impact of the conference. The IAS received input through group consultations, confidential interviews, internet fora, and other meetings. The recommendations from those consultations – among others, to improve the quality of science, to broaden diversity, to facilitate cross-disciplinary linkages and dialogue, and to strengthen the focus on youth – began to be implemented in the planning for the XVI International AIDS Conference (AIDS 2006) in Toronto and will be implemented more fully in planning for the XVII International AIDS Conference (AIDS 2008) in Mexico City. The report from the Future Directions project, including detailed recommendations, is available on the IAS website. We are also strengthening the conference's role as an accountability mechanism, a focus reflected in the conference theme of AIDS 2006, Time to Deliver, that has implications across disciplines and the three programme areas of science, community and leadership.

In addition to the large international conferences, the IAS also organizes the biennial Pathogenesis, Treatment and Prevention conference, which will be held next in Sydney, Australia, 22–25 July 2007. This is the premier open, international scientific meeting on HIV, and was recently expanded to recognize and encourage the importance of biomedical prevention research. It provides researchers and clinicians with opportunities to hear and discuss the latest advances in HIV research across the three major scientific tracks (basic science, clinical science and biomedical prevention science), and to address how advances in research can be rapidly applied to practice. The IAS is committed to continuously improving the conferences and strengthening their role beyond three or five-day events to become part of an ongoing cycle of education, networking, promotion of best practice and advocacy in the fight against HIV/AIDS.

The IAS is also strengthening its commitment to regional development, expanding our involvement in three main areas: collaboration with regional societies, engagement with regional conferences, and supporting and expanding IAS membership in each region. The IAS was a co-organizer of the first Eastern European and Central Asian Conference on AIDS (EECAAC), held 14 – 17 May 2006 in Moscow, and has also provided technical support and advice to other regional conferences including the African regional meeting in Abuja, Nigeria last year. Of course, there is no "one size fits all" approach to regional activities. The unique characteristics of each region require that we build strong relations with key players in each region, learn all we can about the particular issues facing HIV professionals there and tailor our activities to meet the needs and requests for collaboration that we receive.

In addition to its conferences and regional development activities, the IAS coordinates several initiatives. The IAS Industry Liaison Forum is a unique initiative designed to accelerate scientifically promising, ethical research in resource-limited settings, with a particularly focus on the role and responsibility of industry as sponsors and supporters of research. ILF supports dialogue between the pharmaceutical industry, independent investigators and major contributors to research investment such as the Bill & Melinda Gates Foundation. The IAS, under both the ILF and our work with the Bill & Melinda Gates Foundation, has become a significant force in placing pre-exposure prevention prophylaxis (PREP) research on the agenda of policymakers and opinion-leaders. The IAS also co-publishes the electronic Journal of the International AIDS Society (eJIAS) with Medscape General Medicine, a peer-reviewed, open-access scientific journal aimed at publishing research focused on the developing world. Under the leadership of Editor Mark Wainberg (who was recently joined by Ugandan clinician Dr. Elly Katabira as Co-Editor), the volume of submissions to this journal has grown rapidly since its launch in July 2004. It is fully indexed on PubMed and available online [3]. The IAS is also increasingly involved in the development and delivery of a range of education and training programmes designed to help individuals working in HIV advance professionally, from administering the HIV Research Trust Training Scholarships, funded through revenues generated by the International Congress on Drug Therapy in HIV Infection, held biennially in Glasgow, to the development of an IAS Education Programme for IAS 2007. Providing education and training opportunities for its membership is important for any professional society, and the IAS is looking at ways to strategically expand its role in this area in order to strengthen the capacity of the HIV workforce.

The IAS has been increasingly involved in policy and advocacy debates over the past two years. We have advocated strongly for the availability of methadone and buprenorphine in Eastern Europe and Central Asia, where injecting drug use is fuelling that epidemic; on the need for greater political accountability on setting and meeting treatment and prevention targets through the UNGASS process; on increased investment to train, hire and sustain human resources required for scaling up prevention, treatment and care programmes in the developing world. A detailed policy and advocacy strategy, currently in development, will ensure the IAS is an effective advocate for its membership, bringing its voice and influence on key policy issues, in collaboration with other stakeholders engaged in the response to HIV/AIDS.

The role of the IAS as a global knowledge-broker, a facilitator of dialogue, and an independent voice on key HIV/AIDS policy and advocacy issues will strengthen as we move forward in our work in the coming years; the expansion of our membership beyond its traditional base among clinicians and the scientific community requires careful planning in how we develop and deliver training, professional development and networking opportunities to meet the challenges of the coming decades.

The IAS has a unique opportunity – and responsibility – to bring together individuals working in diverse settings and disciplines, from areas of the world separated by geography, culture, language and resources, and to leverage the expertise and knowledge of its members in an effective, sustained global response. If there is a lesson to be learned from our history with this disease, it is that we can do far more collectively than we can individually. As the IAS embarks on an ambitious new phase in its development, we are looking forward to strengthening our engagement with the scientific community, while we build new partnership with NGOs, activists and others working on the frontlines of the response. The IAS encourages all professionals working in the HIV/AIDS field to become members. We are convinced that in the struggle against this epidemic we will be stronger together.

Pedro Cahn, IAS President

Craig McClure, IAS Executive Director
